# Systematic review and meta-analysis of music interventions in hypertension treatment: a quest for answers

**DOI:** 10.1186/s12872-016-0244-0

**Published:** 2016-04-19

**Authors:** Anne Y. R. Kühlmann, Jonathan R. G. Etnel, Jolien W. Roos-Hesselink, Johannes Jeekel, Ad J. J. C. Bogers, Johanna J. M. Takkenberg

**Affiliations:** Department of Pediatric Surgery, Erasmus University Medical Center, Rotterdam, The Netherlands; Department of Cardiothoracic Surgery, Erasmus University Medical Center, P.O. Box 2040, 3000CA Rotterdam, The Netherlands; Department of Cardiology, Erasmus University Medical Center, Rotterdam, The Netherlands; Department of Neuroscience, Erasmus University Medical Center, Rotterdam, The Netherlands

**Keywords:** Hypertension, Music intervention, Meta-analysis, Systemic review, Global health care delivery

## Abstract

**Background:**

Adverse effects, treatment resistance and high costs associated with pharmacological treatment of hypertension have led to growing interest in non-pharmacological complementary therapies such as music interventions. This meta-analysis aims to provide an overview of reported evidence on the efficacy of music interventions in the treatment of hypertension.

**Methods:**

A systematic literature search was conducted for publications on the effect of music interventions on blood pressure in adult hypertensive subjects published between January 1990-June 2014. Randomized controlled trials with a follow-up duration ≥28 days were included. Blood pressure measures were pooled using inverse variance weighting.

**Results:**

Of the 1689 abstracts reviewed, 10 randomized controlled trials were included. Random-effects pooling of the music intervention groups showed a trend toward a decrease in mean systolic blood pressure (SBP) from 144 mmHg(95 % CI:137–152) to 134 mmHg(95 % CI:124–144), and in mean diastolic blood pressure (DBP) from 84 mmHg(95 % CI:78–89) to 78 mmHg(95 % CI:73–84). Fixed-effect analysis of a subgroup of 3 trials with valid control groups showed a significant decrease in pooled mean SBP and DBP in both intervention and control groups. A comparison between music intervention groups and control groups was not possible due to unavailable measures of dispersion.

**Conclusions:**

This systematic review and meta-analysis revealed a trend towards a decrease in blood pressure in hypertensive patients who received music interventions, but failed to establish a cause-effect relationship between music interventions and blood pressure reduction. Considering the potential value of this safe, low-cost intervention, well-designed, high quality and sufficiently powered randomized studies assessing the efficacy of music interventions in the treatment of hypertension are warranted.

**Electronic supplementary material:**

The online version of this article (doi:10.1186/s12872-016-0244-0) contains supplementary material, which is available to authorized users.

## Background

Hypertension has been documented as a major risk factor for cardiovascular morbidity and mortality [1, 2]. Prevalence of hypertension in developed countries is estimated at 37 % and is projected to increase to 42 % by 2025 [3]. When life-style adjustment approaches fail in reducing blood pressure, the main treatment modality in hypertension is pharmacological treatment. Conventional pharmacological treatment is associated with high costs and various adverse effects particularly in cases of combination therapy and treatment resistant hypertension [4]. This has led to a growing interest in non-pharmacological complementary therapies, such as music interventions, in the treatment of hypertension.

Music interventions have been found to affect clinical outcomes in various situations, including short-term effects on blood pressure during medical procedures such as surgery to long-term effects in the treatment of sleep disorders or depression [5–8]. A recent meta-analysis of studies conducted in diverse clinical settings demonstrated that music interventions lead to a significant reduction in systolic blood pressure (SBP), diastolic blood pressure (DBP) and heart-rate in various disease states [9]. Another review found that listening to music may have a beneficial effect on anxiety, SBP, heart-rate, respiratory rate, quality of sleep and pain in patients with coronary heart disease [10].

Music interventions can be administered in different ways. They can be either live or recorded and administered either with or without the involvement of a music therapist. Moreover, the music intervention can be chosen by the patient, by a music therapist or by a healthcare practitioner – the latter especially in the case of research. There are various definitions of music-based interventions, such as ‘music therapy’, ‘receptive music’ and ‘music medicine’. According to the definition of the American Music Therapy Association, music therapy is the clinical and evidence-based use of music interventions to accomplish individualized goals within a therapeutic relationship by a credentialed professional who has completed an approved music therapy program [11]. The therapeutic relationship is an important aspect in this definition. The term ‘receptive music’ is meant as a broader explanation of music-based interventions and encompasses several techniques in which the client is a recipient of the music experience [12]. It may also be part of a therapeutic relationship. Another definition is music medicine and can either refer to selected and often specially composed music which is thought to have an effect itself [13] or can be defined as passive listening to prerecorded music provided by medical personal other than a music therapist [14].

Several studies have been performed to examine the possible effects of music on hypertension. These studies are usually small in sample-size and an overview of reported outcomes is lacking. To investigate the potential anti-hypertensive effect of music interventions, we conducted a systematic review and meta-analysis of prospective randomized controlled trials that assessed the effect of music interventions on blood pressure in hypertensive patients.

In this article, we describe the effects of several types of music interventions in patients with hypertension. Overall, we will use the broader term music interventions. However when we specifically differentiate the interventions, we will refer to music therapy when a specific intervention includes the involvement of a music therapist in a therapeutic relationship. Music interventions without this therapeutic relationship will be referred to as recorded music interventions.

## Methods

This systematic review was conducted according to the PRISMA guidelines [15]. The study was approved by the institutional review board (MEC 2014–384) and informed consent was waived. On June 6th, 2014 Embase, PubMed, Medline, Cochrane Central, Web of Science and Google Scholar were searched for publications on the effect of music on blood pressure in adult hypertensive patients (see Additional file 1). Results were screened manually on relevance by two independent investigators (AYRK, JRGE). Studies on the effect of music interventions on blood pressure in hypertensive patients with mean age ≥18 years were considered for inclusion. Studies conducted in humans, published after 1/1/1990, written in English, German, French, Dutch, or Spanish and with a follow-up of at least 28 days were included. Studies were excluded if the full text was not available. Cohorts that received any additional treatment other than music and/or standard medical therapy were also excluded. Cohorts with a medical history of hypertension, with or without medical treatment, or a mean SBP ≥ 140 mmHg and/or DBP ≥ 90 mmHg at baseline were included [1]. Music interventions had to be administered multiple times during the trial period. There were no limitations on the type of music administered, nor on the timing of each intervention.

Methods of analysis and inclusion criteria were specified and documented in advance. Only the most recent or most complete study was included if there was an overlap in study populations. In case of disagreement on the inclusion of a paper, an agreement was negotiated.

### Data extraction & statistical analyses

Microsoft Office Excel 2011 (Microsoft Corp., Redmond, WA, USA) was used for data extraction and statistical analyses. The following patient and study characteristics were recorded: age, sex, systolic and diastolic blood pressure at baseline, history of hypertension, use of antihypertensive medication, comorbidities, details of music intervention and length of follow-up. Primary outcome measures were reduction in SBP and DBP and mean SBP and DBP at last follow-up. Secondary outcome measures were effects of music on anxiety and quality of life.

Weighted pooling was conducted on the patient characteristics. Mean SBP and DBP at baseline and at final follow up and mean reduction in SBP and DBP were pooled using inverse variance weighting in a random-effects model. When the number of studies was not sufficiently large to reliably estimate the tau-squared statistic (<4 studies), a fixed-effect model was used as well [16]. Studies that did not provide any measure of dispersion for the mean of a particular variable were excluded from the meta-analysis of that variable. Heterogeneity among the included studies was analyzed with both the Cochran Q statistic and the I2 index. Risk of bias among studies was assessed using the Cochrane Collaboration risk of bias assessment tool [17]. Funnel plots were used to investigate publication bias. Statistical significance was inferred at a *p*-value <0.05.

## Results

**Fig. 1:**
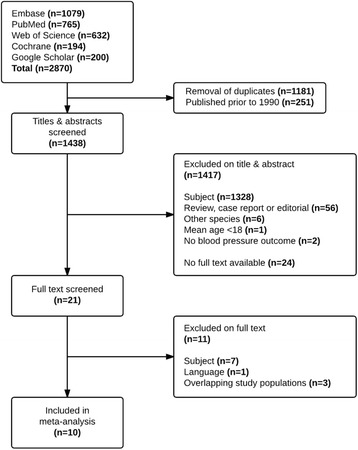
Flowchart of literature search and study selection

The literature search resulted in 1689 publications. Ten of these studies, encompassing a total of 296 patients, met all of the described criteria and were included in the systematic review (Fig. 1) [2, 18–26]. All of these were randomized controlled trials published in English. Table 1 provides an overview of the included studies and baseline patient characteristics.

**Table 1:** Overview of publications, all RCTs

First author	Year	Study arm	*N*	Age (years)	M %	History of HT %	Anti-HT drugs %	Follow-up (days)	Timing of intervention	Description
Bekiroglu [18]^a^	2013	M	30	75.5 (7.1)	57	100	90	28	1×/day	Turkish Classical
		C	30	78.2 (6.1)	57	100	90			Resting Period
Modesti [2]	2010	M	26	58.0 (−)	62	100	77	180	1×/day	Classical, Celtic, Indian
		C	29	58.0 (−)	55	100	73			Music Guided Slow-Breathing (Buteyko-Pranayama)
Zanini [19]^a^	2009	M	23	66.5 (9.1)	30	100	100	84	1×/week	Recreation, Improvisation, Composition, Listening of music^b^
		C	22	67.2 (9.6)	55	100	100			No Intervention (Standard Medical Therapy)
Chan [20]^a^	2009	M	23	>60	44	61	-	28	1×/day-1×/week	Western-, Chinese-, Asian Classical, Western Jazz
		C	24	>60	46	67	-			Resting Period
Tang [25]	2009	M	22	85.0 (5.0)	18	59	32	84	3×/week	Mozart
		C	19	86.0 (6.0)	11	68	42			Audio Relaxation Program Training (Revitalizer II)
Altena [24]	2009	M	15	59.0 (11.7)	53	100	100	63	Preferably daily	Slow Music
		C	15	60.0 (11.0)	47	100	100			Resperate® (Device Guided Breathing Exercises)
Pandic [22]	2008	M	22	66.5 (8.3)	18	100	77	112	3×/week	Relaxing Music
		C	31	70.4 (8.7)	32	100	77			Resperate® (Device Guided Breathing Exercises)
Logtenberg [23]	2007	M	15	59.0 (11.7)	67	100	100	56	1×/day	Various Kinds of Music
		C	15	62.7 (6.0)	20	100	100			Resperate® (Device Guided Breathing Exercises)
Schein [21]	2001	M	29	56.5 (8.0)	39	100	76	56	1×/day	Quiet Synthesized Music with Non-Identifiable Rhythm
		C	32	57.8 (9.4)	56	100	91			Breathe with Interactive Music (BIM) Device
Grossman [26]	2001	M	15	50.0 (4.0)	67	100	53	56	1×/day	Quiet Synthesized Music with Non-Identifiable Rhythm
		C	18	52.0 (12.0)	72	100	56			Breathe with Interactive Music (BIM) Device

*RCT* randomized controlled trial, *M* music arm, *C* control arm, *HT* hypertension

^a^Study with Resting period/No intervention as control group

^b^This study used music therapy whereas the others used recorded music interventions

Nine studies evaluated the effects of recorded music interventions whereas one study evaluated the effects of music therapy [19]. There was a large variation in follow-up duration and in the type, timing and duration of music intervention sessions among the included studies. Seven of the 10 included studies compared music interventions to various other interventions and, thus, did not allow for comparative analysis. Mean age of the patients in the music intervention arms was 65.2 ± 7.3 years and 42 % were male. A medical history of hypertension was reported in 92 % of the patients and 78 % used anti- hypertensive drugs.

Only three studies reported prevalence of comorbidities, such as respiratory disease or diabetes mellitus, which varied from 26 to 100 % in their cohorts [18, 20, 23]. Overall, there was a moderate to high risk of bias among the included studies (see Additional file 2) [17]. Due to the small variation in sample size of the included studies, analysis of publication bias was inconclusive (see Additional file 3).

### Music interventions and blood pressure

Table 2 shows blood pressure data of the music intervention arms of all ten included studies, pooled in a random-effects model. In the pooled analysis of mean SBP and DBP at baseline and last follow-up, music interventions were associated with a decrease in SBP from 144 mmHg to 134 mmHg, as well as a decrease in DBP from 84 mmHg to 78 mmHg. Pooling of the mean *reduction* in blood pressure in each study also showed a reduction in both systolic and diastolic blood pressure after music interventions, though due to unavailable measures of dispersion, five of the studies were excluded from this analysis. Strong evidence of heterogeneity was observed among all outcome measures.

**Table 2:** Pooled outcome measures of music intervention arms of included studies

First author	SBP baseline (mmHg)	SBP end (mmHg)	DBP baseline (mmHg)	DBP end (mmHg)	Mean SBP reduction (mmHg)	Mean DBP reduction (mmHg)
Bekiroglu [18]	128.2 (6.7)	115.2 (5.3)	77.5^a^(−)	70.0^a^(−)	13.0^a^(−)	7.5^a^(−)
Modesti [2]	131.0 (13.0)	129.7^a^(−)	79.0 (9.1)	77.6^a^(−)	1.3 (7.0)	1.4 (5.4)
Zanini [19]	149.7 (6.4)	133.8 (13.4)	89.1 (9.1)	80.1 (10.6)	15.9^a^(−)	9.0^a^(−)
Chan [20]	143.8 (23.8)	130.1 (28.1)	73.1 (11.5)	67.7 (14.0)	17.3^a^(−)	5.4^a^(−)
Tang [25]	145.0 (19.0)	139.0 (17.0)	74.0 (10.0)	71.0 (10.0)	6.0^a^(−)	3.0^a^(−)
Altena [24]	133.9 (15.7)	131.0 (11.5)	78.4 (11.1)	75.0 (13.2)	2.9 (6.1)	3.4 (9.2)
Pandic [22]	151.8 (15.7)	135.1 (10.6)	82.7 (9.8)	78.7 (7.7)	16.0^a^(−)	4.1^a^(−)
Logtenberg [23]	150.4 (8.2)	138.2 (10.3)	87.0 (8.3)	81.5 (8.3)	12.2 (9.4)	5.5 (7.5)
Schein [21]	154.7 (8.5)	143.4^a^(−)	93.4 (7.1)	87.8^a^(−)	11.3 (12.8)	5.6 (6.2)
Grossman [26]	155.0 (11.0)	152.1 (12.1)	94.0 (6.0)	92.5 (9.1)	2.9 (12.1)	1.5 (9.1)
R-E model	144.4	134.3	83.6	78.2	6.0	3.5
(95 % CI)	(136.7–152.1)	(124.0–144.5)	(78.2–88.9)	(72.6–83.8)	(1.5–10.4)	(1.4–5.7)
Heterogeneity	*X* ^2^ *P* < 0.001	*X* ^2^ *P* < 0.001	*X* ^2^ *P* < 0.001	*X* ^2^ *P* < 0.001	*X* ^2^ *P* < 0.001	X^2^ *P* = 0.061
	I^2^ = 97 %	I^2^ = 97 %	I^2^ = 95 %	I^2^ = 91 %	I^2^ = 84 %	I^2^ = 56 %

Data expressed as “mean (SD)”, “mean (95 % CI)” or proportions

*R-E model* random-effects model, *SBP* systolic blood pressure, *DBP* diastolic blood pressure

^a^Excluded from analysis due to unavailable measures of dispersion

### Music interventions versus standard care

**Fig. 2:**
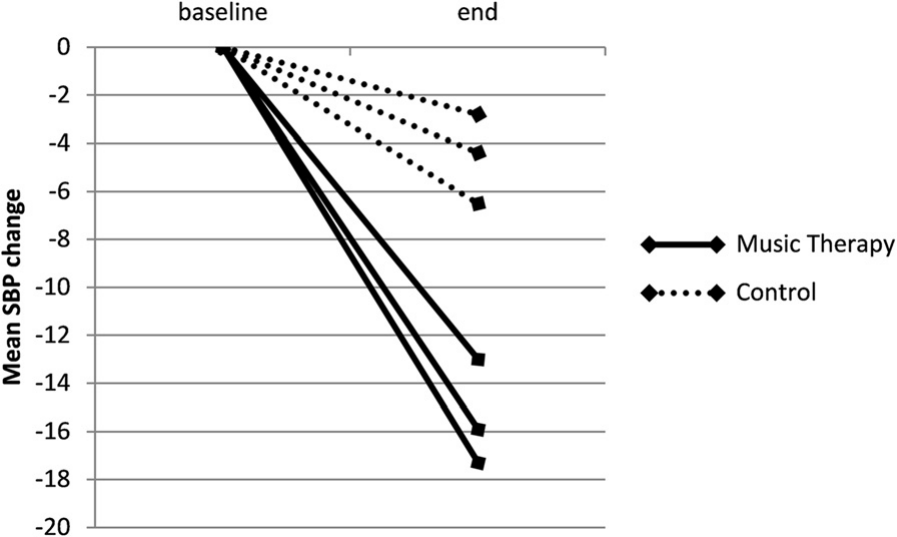
Mean change in systolic blood pressure in different study-arms in the three comparative studies. SBP = systolic blood pressure

**Fig. 3:**
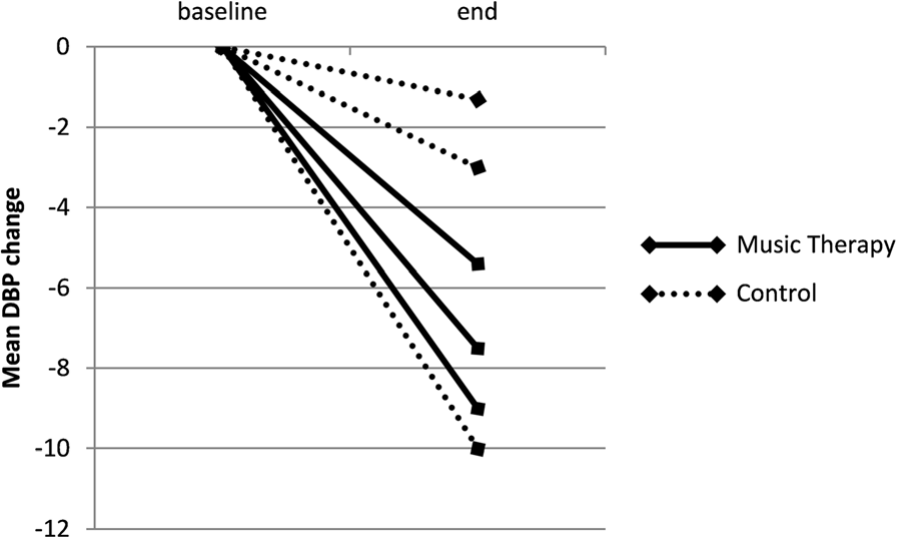
Mean change in diastolic blood pressure in different study-arms in the three comparative studies. DBP = diastolic blood pressure

Three of the ten included studies compared music interventions to a control group that received either standard medical therapy or a resting period [18–20]. Mean age of the patients in the control groups was 73.6 ± 7.8 years and 53 % were male. A medical history of hypertension was reported in 89 % of the patients. When comparing pooled mean SBP/DBP at baseline with pooled mean SBP/DBP at the end of the trial period in a random-effects model, a trend towards a decrease was found in pooled mean SBP and DBP in treatment as well as control groups, while fixed-effect analysis showed a significant decrease in both groups (Table 3). None of these 3 trials made a formal comparison of the observed reduction in blood pressure between the treatment and control groups. Although the magnitude of this reduction appeared to be greater in the experimental groups when represented graphically (Figs. 2 and 3), due to unavailable measures of dispersion a formal comparison of the mean *reduction* in SBP and DBP between the music interventions- and control group was not possible in this subgroup analysis.

**Table 3:** Pooled outcome measures of the studies with both intervention and control arms

		SBP baseline (mmHg)	SBP end (mmHg)	DBP baseline (mmHg)	DBP end (mmHg)
Intervention	Bekiroglu [18]	128.2 (6.7)	115.2 (5.3)	77.5^a^(−)	70.0^a^(−)
	Zanini [19]	149.7 (6.4)	133.8 (13.4)	89.1 (9.1)	80.1 (10.6)
	Chan [20]	143.8 (23.8)	130.1 (28.1)	73.1 (11.5)	67.7 (14.0)
Heterogeneity		*X* ^2^ *P* < 0.001	*X* ^2^ *P* < 0.001	*X* ^2^ *P* < 0.001	*X* ^2^ *P* < 0.001
		I^2^ = 99 %	I^2^ = 96 %	I^2^ = 96 %	I^2^ = 91 %
R-E model		140.4	126.0	81.2	74.1
(95 % CI)		(123.7–157.2)	(111.5–140.5)	(65.5–96.9)	(61.9–86.2)
F-E model		138.2	117.5	82.9	75.6
(95 % CI)		(136.5–140.0)	(115.7–119.2)	(80.0–85.9)	(72.1–79.0)
Control	Bekiroglu [18]	121.2 (5.9)	114.7 (6.0)	80.0^a^(−)	70.0^a^(−)
	Zanini [19]	145.4 (5.6)	141.0 (19.8)	86.9 (11.3)	83.9 (12.4)
	Chan [20]	143.7 (22.1)	140.9 (26.4)	72.7 (12.8)	71.4 (13.6)
Heterogeneity		*X* ^2^ *P* < 0.001	*X* ^2^ *P* < 0.001	*X* ^2^ *P* < 0.001	*X* ^2^ *P* = 0.001
		I^2^ = 99 %	I^2^ = 96 %	I^2^ = 94 %	I^2^ = 91 %
R-E model		136.6	131.8	79.8	77.7
(95 % CI)		(117.6–155.6)	(110.9–152.7)	(65.9–93.8)	(65.4–89.9)
F-E model		132.5	117.3	80.4	78.0
(95 % CI)		(130.9–134.0)	(115.2–119.3)	(76.9–83.9)	(74.2–81.7)

Data expressed as “mean (SD)”, “mean (95 % CI)” or proportions

*R-E model* random-effects model, *F-E model* fixed-effect model, *SBP* systolic blood pressure, *DBP* diastolic blood pressure

^a^Excluded from analysis due to unavailable measures of dispersion

### Anxiety and quality of life

Five studies evaluated the effects of the music intervention on quality of life and anxiety [2, 18, 19, 23, 24]. One study found significant improvements in quality of life [19]. This finding was not supported by the other studies. Due to the large variety of questionnaires used in these studies, pooling of these results was not possible.

## Discussion

This systematic review and meta-analysis of ten randomized controlled trials evaluating the effect of music interventions in the treatment of hypertension found a decrease in pooled mean SBP and DBP after application of music interventions, however this decrease did not reach statistical significance. In the subgroup of three studies with a standard medical therapy or resting control group, random-effects analysis revealed a trend towards a blood pressure decrease in both the intervention and the control groups, while fixed-effect analysis showed a significant decrease in both groups [18–20].

Unfortunately, a valid comparison between the music intervention- and control group did not prove possible, and a cause-effect relationship between music interventions and hypertension remains to be determined.

Research has shown that relatively small decreases, as low as 5 mmHg reduction in systolic blood pressure, would result in 7 % reduction in all-cause mortality, 9 % reduction in coronary heart disease related mortality and 14 % reduction in stroke-related mortality [1, 27]. These numbers illustrate the substantial benefit of even small decreases in blood pressure, and if indeed in future studies music interventions prove to be effective, it would provide a valuable low cost therapeutic measure.

The mechanism by which music modulates blood pressure remains unclear. Studies on device-guided breathing hypothesize that reduction in blood pressure is achieved by modulating autonomous cardiovascular regulation by slowing down the breathing frequency [2, 21–25]. As a result, baroreflex sensitivity is lowered, parasympathetic tonus increases and sympathetic tonus decreases, resulting in a decrease in blood pressure. Music listening might elicit the same relaxation response, resulting in a decrease in blood pressure. Another possible mechanism of action is that music interventions lead to increased brain dopamine levels via a calmodulin-dependent system. This increase in dopamine levels inhibits sympathetic activity via dopamine-2 receptors which in turn reduces blood pressure [28].

Furthermore, music may direct one’s attention to a more pleasant emotional state, thereby triggering feelings associated with physical and mental relaxation [29]. It might also give rise to positive emotions which are connected with the activation of the limbic system, thereby releasing endorphins affecting physiological systems [30]. Moreover, a recent review on magnitude of blood pressure reduction in the placebo arms of hypertension trials found a significant pooled blood pressure reduction of 6 mmHg after placebo intervention [31]. This non-trivial placebo effect should be taken into account when offering these patients any treatment.

The random-effects subgroup analysis of the three trials with comparable control groups showed a trend towards a decrease in blood pressure in both the intervention- and control groups [18–20]. Although a random-effects model may be most appropriate in this case in light of the substantial heterogeneity among these studies, the small number of studies makes quantitative estimation of the between-study variance in this subgroup very unreliable. We therefore chose to apply a fixed-effect model to this subgroup as well, which revealed a significant decrease in blood pressure in both intervention- and control groups. However, this fixed-effect analysis does not take the considerable heterogeneity that we observed into account. Thus, in the case of this subgroup, the inherent limitations of both methods renders these analyses inconclusive and the results should be interpreted with caution.

The observed blood pressure decrease in the control groups of this subgroup analysis may be explained in part by the fact that in two of these studies the patients were prescribed a resting period as control, possibly eliciting autonomic responses similar to those described above [18, 20]. When visually assessing the mean *reduction* in SBP and DBP in each of these studies, the magnitude of this reduction appeared to be greater in the experimental groups, however a formal comparison of pooled mean *reduction* of blood pressure between the music and control group was not possible due to missing measures of dispersion concerning this reduction. As a result, a cause-effect relationship could not be established and the only conclusion to be drawn from our meta-analysis, is that we observed a significant decrease in blood pressure in hypertensive patients who underwent music interventions, but also in control patients. These observations could simply be the result of regression toward the mean.

Prevalence of co-morbidities, such as respiratory disease or diabetes mellitus, varied from 26 to 100 % in the three studies that reported it [18, 20, 23]. Data on association of co-morbidities and response to music interventions were not available from these studies. The presence of comorbidities, but also etiology of hypertension, treatment resistance and possible seasonal effects could potentially influence the effect of an intervention [31, 32]. These aspects should be taken into account when evaluating the effect of the intervention.

### Anxiety, depression and quality of life

Zanini et al. was the only study that found an association between music interventions and quality of life, which might be explained by the use of music therapy in their study in contrast to recorded music interventions in the other studies [19]. Although recorded music interventions were found to be as effective as music therapy in reducing periprocedural pain and anxiety in children undergoing medical procedures [33], it is likely that the effect of music interventions in other settings may indeed be influenced by the method of administration. In some disease states, for instance in psychological or psychiatric disorders or rehabilitation, the involvement of a credentialed music therapy professional may provide better results than listening to music without a music therapist. Furthermore, the difference in effectiveness of music therapy compared with recorded music interventions may depend not only on the disease state, but also on which outcome is studied. Improvement of quality of life might be an outcome where dedicated involvement of a therapist providing personalized care may yield greater improvement than solely listening to music. Pain relief on the other hand, may be more strongly regulated by mechanisms triggered by both music therapy and recorded music interventions, such as redirecting someone’s attention or activation of the limbic system and the subsequent release of endorphins. Pain relief, in contrast to improvement of quality of life, may therefore be less dependent on involvement of a music therapist. Furthermore, the variation in results concerning quality of life among the included studies might also be explained by the shorter duration of some studies and the difference in study populations.

As for anxiety, Bekiroglu et al. found no significant effects of music interventions [18]. As they suggest, this may be explained by the lack of high anxiety levels at baseline in their patient population, as most likely may be the case in the hypertensive patient population at large. Music interventions might be more effective in decreasing anxiety when patients face a more challenging condition causing extensive anxiety, such as patients suffering from myocardial infarction or facing surgery [6, 10, 34, 35].

### Music intervention variability

A major complicating factor in our analysis of music interventions was the large variation in the type of music administered and the frequency and duration of interventions in the included studies (Table 1). Although the majority of interventions included classical, relaxing or slow music, no clear recommendations exist on how music interventions should be administered in the treatment of high blood pressure. A systematic review on music interventions in anxiety and pain relief in clinical practice provide some insights on which music may be most beneficial [36]. The authors recommend patient-preferred slow and flowing music, approximately 60 to 80 beats per minute, with a minimum duration of 30 min in length. Research in hypertensive animal models found music containing high-frequency sounds to stimulate dopamine synthesis leading to blood pressure reduction [37]. Moreover, music interventions may be greatly enhanced by preference and familiarity of the patients. Anxiety- and pain reducing effects appear to be greatest when people are given a choice of music to listen to or listen to their own favorite music and other research suggests patient-preferred music, as opposed to prescribed music, to be a critical factor in the effectiveness of music interventions [5, 10, 35, 38]. The observed large variation in the types of music used, the applications of music interventions, and the outcomes studied, illustrate the complexity of the topic, and pose a major challenge for future studies.

### Limitations

As with any meta-analysis, the general limitations inherent to meta-analyses should be taken into account [39]. Since the number of patients included in each study is very small and no formal comparison of the treatment effect between the music intervention- and control group was possible, no hard conclusions can be drawn concerning the effect of music interventions on hypertension. As described above, the inherent limitations of both fixed- and random-effects models in the case of a very small, heterogeneous sample of studies rendered our subgroup analysis inconclusive. There was significant heterogeneity in the reported outcomes, which is most likely the result of the large methodological variation among the included studies with regard to patient characteristics, the type of music administered, the duration of each intervention and the follow-up time.

Randomization was mentioned in all trials, though specific information on trial conduct, such as allocation concealment and blinding, was reported poorly and therefore quality assessment of the included studies was limited (see Additional file 2). This, as well as incomplete outcome data, gave rise to a moderate to high risk of bias in the included studies. Publication bias may have affected the outcomes, as some abstracts were unavailable as full-text articles (Fig. 1).

### Perspectives

Our results show that current studies on the effect of music interventions on lowering blood pressure in hypertensive patients do not provide evidence on a possible cause-effect relationship. Since music interventions may be of beneficial value in hypertensive patients, presenting a potential adjuvant to standard pharmacological treatment, there is a need for further high quality research on the subject. Music interventions could not only be of value in case of multidrug therapy or treatment resistant hypertension, but might also be offered as a durable treatment modality in developing countries. However, well-designed high-quality, sufficiently powered randomized controlled trials are first required to establish a cause-effect relationship between music interventions and blood pressure reduction in hypertensive patients.

This research, ideally in the form of large, well-reported randomized controlled trials following the CONSORT statement for nonpharmacological trials with clearly-defined interventions and controls and adequate statistical analyses, could explore the ability of music interventions in lowering blood pressure in a large population, examine the permanence of the reduction in blood pressure and elucidate which patients could benefit most [40]. The influence of different forms of music intervention, with regard to factors such as genre and patient-preference, should be investigated. In addition both music therapy and recorded music interventions could be analyzed to obtain more knowledge on the manner of administration of music interventions in the treatment of hypertension. Finally, evaluation of factors that may play a role in the sensitivity to corrections of elevated blood pressure, such as baroreflex sensitivity, can be explored.

## Conclusion

This systematic review and meta-analysis found a trend towards a decrease in blood pressure in hypertensive patients who received music interventions. Unfortunately, this decrease does not provide proof for a cause-effect relationship, as a formal comparison with the control group is lacking. Therefore the most important conclusion of this study is that the quest for answers is still ongoing. Considering the potential value of this safe, low-cost intervention, there is an urgent need for well-designed, high quality, sufficiently powered randomized studies that assess the efficacy of music interventions in lowering blood pressure.

### Availability of data and materials

The datasets supporting the conclusions of this article are included within the article (and its additional files).

## Additional files

Additional file 1: Literature search. (PDF 64.9 kb) Additional file 2: Risk of bias assessment. (PDF 34 kb) Additional file 3: Funnel plots. (PDF 61.9 kb)

## Abbreviations

DBP: diastolic blood pressure
SBP: systolic blood pressure
