# Supplementary material for: Systematic review and meta-analysis of music interventions in hypertension treatment: a quest for answers
Source: BMC Cardiovasc Disord. 2016 Apr 19;16:69. doi: 10.1186/s12872-016-0244-0 (PMC4837643; doi:10.1186/s12872-016-0244-0)
Supplement: Additional file 1: — Literature search. (PDF 64.9 kb) [file 12872_2016_244_MOESM1_ESM.pdf]

Additional file 1. Literature Search.

Embase.com 1079 results
(music/de OR 'acoustic stress'/de OR 'music therapy'/de OR singing/de OR musician/de OR 'auditory stimulation'/de OR 'MP3 player'/de OR 'tape recorder'/de OR 'compact disk'/de OR (music* OR melod* OR song* OR ((audi* OR acoustic* OR sound*) NEAR/6 (stimul* OR stress)) OR mp3 OR earphone* OR headphone* OR ((ear OR head) NEXT/1 phone*) OR 'compact disk' OR ((cd OR cassette) NEXT/1 player*) OR speaker*):ab,ti) AND ('abnormal blood pressure'/de OR 'elevated blood pressure'/exp OR 'blood pressure measurement'/exp OR 'blood pressure meter'/exp OR 'blood pressure'/exp OR (hypertens* OR ((blood OR arter* OR diastol* OR systol*) NEAR/3 pressure) OR sphygmomanomet*):ab,ti) NOT ([animals]/lim NOT [humans]/lim)

Medline (ovidSP) 751 results
(music/ OR "music therapy"/ OR singing/ OR "Acoustic Stimulation"/ OR "MP3-player"/ OR "Tape Recording"/ OR Radio/ OR "Compact Disks"/ OR (music* OR melod* OR song* OR ((audi* OR acoustic* OR sound*) ADJ6 (stimul* OR stress)) OR mp3 OR earphone* OR headphone* OR ((ear OR head) ADJ phone*) OR "compact disk" OR ((cd OR cassette) ADJ player*) OR speaker*).ab,ti.) AND (exp "Hypertension"/ OR "Blood Pressure Determination"/ OR exp "blood pressure"/ OR (hypertens* OR ((blood OR arter* OR diastol* OR systol*) ADJ3 pressure) OR sphygmomanomet*).ab,ti.) NOT (exp animals/ NOT humans/)

Cochrane 194 results
((music* OR melod* OR song* OR ((audi* OR acoustic* OR sound*) NEAR/6 (stimul* OR stress)) OR mp3 OR earphone* OR headphone* OR ((ear OR head) NEXT/1 phone*) OR 'compact disk' OR ((cd OR cassette) NEXT/1 player*) OR speaker*):ab,ti) AND ((hypertens* OR ((blood OR arter* OR diastol* OR systol*) NEAR/3 pressure) OR sphygmomanomet*):ab,ti)

Web-of-science 632 results
TS=(((music* OR melod* OR song* OR ((audi* OR acoustic* OR sound*) NEAR/6 (stimul* OR stress)) OR mp3 OR earphone* OR headphone* OR ((ear OR head) NEAR/1 phone*) OR "compact disk" OR ((cd OR cassette) NEAR/1 player*) OR speaker*)) AND ((hypertens* OR ((blood OR arter* OR diastol* OR systol*) NEAR/3 pressure) OR sphygmomanomet*)))

PubMed publisher 14 results
((music*[tiab] OR melod*[tiab] OR song*[tiab] OR ((audi*[tiab] OR acoustic*[tiab] OR sound*[tiab]) AND (stimul*[tiab] OR stress)) OR mp3[tiab] OR earphone*[tiab] OR headphone*[tiab] OR ear phone*[tiab] OR head phone*[tiab] OR compact disk*[tiab] OR cd player*[tiab] OR cassette player*[tiab] OR speaker*[tiab])) AND (((blood[tiab] OR arter*[tiab] OR diastol*[tiab] OR systol*[tiab]) AND pressure[tiab]) OR sphygmomanomet*[tiab])) AND publisher[sb]

Google scholar 200 results
Music|singing|musician|"auditory stimulation "blood pressure"|hypertension
