# Supplementary material for: Systematic review and meta-analysis of music interventions in hypertension treatment: a quest for answers
Source: BMC Cardiovasc Disord. 2016 Apr 19;16:69. doi: 10.1186/s12872-016-0244-0 (PMC4837643; doi:10.1186/s12872-016-0244-0)
Supplement: Additional file 2: — Risk of bias assessment. (PDF 34 kb) [file 12872_2016_244_MOESM2_ESM.pdf]

Additional file 2. Risk of Bias Assessment.

	Sequence generation	Allocation sequence concealment	Blinding of participants*	Blinding of health care providers	Blinding of data collectors	Blinding of outcome assessment	Incomplete outcome data	Other potential sources of bias
Bekiroglu (2013)	Adequate	Unclear	Inadequate	Unclear	Inadequate	Adequate	No missing data	-
Modesti (2010)	Unclear	Unclear	Inadequate	Adequate	Unclear	Adequate	2 missing data	-
Zanini (2009)	Adequate	Unclear	Inadequate	Unclear	Unclear	Adequate	1 missing data	-
Chan (2009)	Adequate	Adequate	Inadequate	Inadequate	Inadequate	Adequate	3 missing data, imputation of mean	Funding by School of Nursing of Hong Kong Polytechnic University (A-PH29)
Tang (2009)	Adequate	Unclear	Inadequate	Unclear	Unclear	Adequate	2 missing data	Funding John J. Locke Jr. Charitable Trust, Perpetuity
Altena (2009)	Adequate	Adequate	Inadequate	Unclear	Unclear	Adequate	No missing data, ITT analysis	Financial support Medical Research Foundation Zwolle
Pandic (2008)	Adequate	Adequate	Inadequate	Unclear	Unclear	Unclear	1 missing data	Grant Health & Medical Care Committee Göteborg & Bohuslän
Logtenberg (2007)	Adequate	Unclear	Inadequate	Unclear	Unclear	Adequate	No missing data, ITT analysis	Financial support Medical Research Foundation and Langerhans Foundation
Schein (2001)	Adequate	Unclear	Inadequate	Adequate	Adequate	Adequate	4 missing data	Sponsored by InterCure Ltd, Chief Scientist is last author
Grossman (2001)	Unclear	Inadequate	Inadequate	Adequate	Adequate	Adequate	1 patient changed study-arm	Sponsored by InterCure Ltd, Chief Scientist is last author

* Blinding of participants was judged as inadequate in all studies as participants cannot be blinded for music-interventions. ITT=Intention To Treat.
