# Supplementary material for: Systematic review and meta-analysis of music interventions in hypertension treatment: a quest for answers
Source: BMC Cardiovasc Disord. 2016 Apr 19;16:69. doi: 10.1186/s12872-016-0244-0 (PMC4837643; doi:10.1186/s12872-016-0244-0)

Additional file 3. Funnel plots

Legend: Funnel plots of mean reduction in blood pressure against each study's precision or size. Because of missing measures of dispersion in 5 out of 10 studies, plotting was also performed against study size.

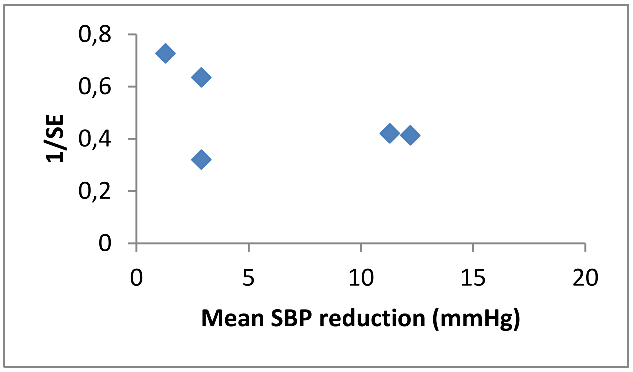

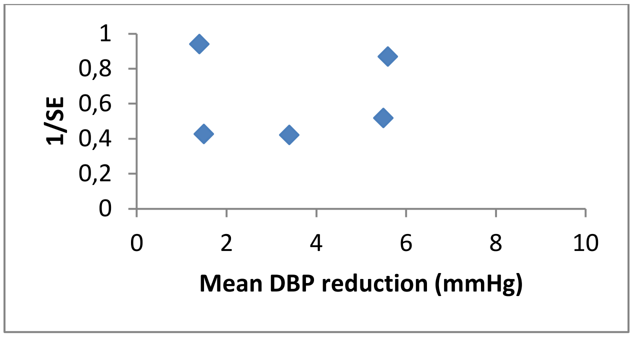

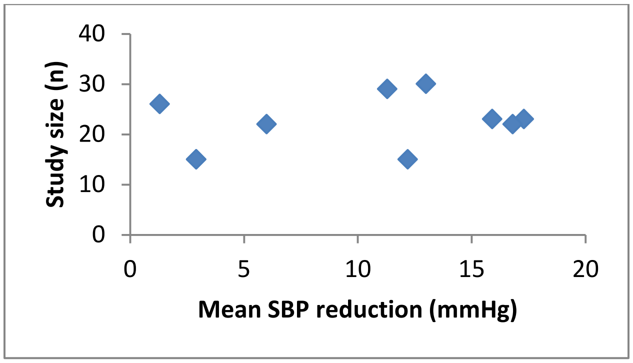

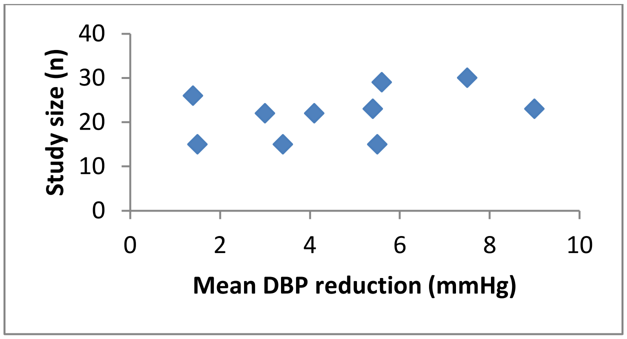

Supplement: Additional file 3: — Funnel plots. (PDF 61.9 kb) [file 12872_2016_244_MOESM3_ESM.pdf]
